# Exosomal circHIPK3 Released from Hypoxia-Pretreated Cardiomyocytes Regulates Oxidative Damage in Cardiac Microvascular Endothelial Cells via the miR-29a/IGF-1 Pathway

**DOI:** 10.1155/2019/7954657

**Published:** 2019-12-05

**Authors:** Yan Wang, Ranzun Zhao, Weiwei Liu, Zhenglong Wang, Jidong Rong, Xianping Long, Zhijiang Liu, Junbo Ge, Bei Shi

**Affiliations:** ^1^Department of Cardiology, Affiliated Hospital of Zunyi Medical University, Zunyi 563000, China; ^2^Department of Cardiology, Shanghai Institute of Cardiovascular Diseases, Zhongshan Hospital, Fudan University, Shanghai 200032, China

## Abstract

**Background/Aims:**

Circular RNAs (circRNAs) are a class of endogenous noncoding RNAs that regulate gene expression in eukaryotes. Recently, exosomes from cardiomyocytes (CMs) have been found to facilitate cell proliferation and survival by transporting various bioactive molecules, including circRNA. However, the functions of exosomal circRNAs are not clear. The present research is aimed at determining whether circHIPK3 released from hypoxia-pretreated CMs is transferred into cardiac microvascular endothelial cells (CMVECs) by exosomes and becomes functionally active in the CMVECs under oxidative stress conditions.

**Methods:**

Quantitative polymerase chain reactions were conducted to detect the expression pattern of circHIPK3 in CMVECs under oxidative stress. Annexin V-FITC/propidium iodide (PI) staining assays, TUNEL assays, ROS assays, and Western blot analysis were conducted to detect the role of exosomal circHIPK3 in CMVEC function in vitro. Luciferase activity assays and RNA immunoprecipitation studies were conducted in vitro to reveal the mechanism of circHIPK3-mediated CMVEC function.

**Results:**

circHIPK3 expression was significantly upregulated in hypoxic exosomes (HPC-exos) compared with normoxic exosomes (Nor-exos). Moreover, HPC-exos induced stronger antioxidant effects than Nor-exos. The silencing or overexpression of circHIPK3 changed CMVEC survival under oxidative conditions in vitro. Furthermore, circHIPK3 silencing in HPC-exos abrogated the protective effects of HPC-exos in CMVECs, as shown by increased levels of apoptosis, ROS, MDA, and proapoptotic proteins. circHIPK3 acted as an endogenous miR-29a sponge to sequester and inhibit miR-29a activity, which led to increased IGF-1 expression. The ectopic expression of miR-29a mimicked the effect of circHIPK3 silencing in CMVECs in vitro.

**Conclusions:**

circHIPK3 in HPC-exos plays a role in CMVECs under oxidative conditions through miR-29a-mediated IGF-1 expression, leading to a decrease in oxidative stress-induced CMVECs dysfunction. These data suggest that the exosomal circRNA in CMs is a potential target to control CMVECs dysfunction under oxidative conditions.

## 1. Introduction

Microcirculatory dysfunction is an important etiological component of ischemia-reperfusion injury [1]. Oxidative stress caused by a surge in the generation of reactive oxygen species (ROS) during reoxygenation can disrupt microvascular integrity [2], consequently decreasing the oxygen and nutrients supplied to cardiac cells. Cardiac microvascular endothelial cells (CMVECs) play an obligatory role in regulating and maintaining cardiac function by forming connections and constituting the continuous endothelium between the circulation and cardiomyocytes (CMs) [3, 4]. The response of CMVECs to ROS impacts heart function via changes in endothelial barrier function that subsequently disrupt tissue blood flow. To ensure sufficient blood supply to deprived areas [5], it is important to explore powerful strategies to protect CMVECs from oxidative stress. The maintenance of microvascular anatomic and functional integrity after ischemia-reperfusion injury is a highly controlled mechanism that involves communication between the different cell types in the heart [6]. Typically, some level of direct communication is established between CMs and CMVECs. The close contact between CMs and CMVECs allows for the transfer of oxygen and metabolic information from CMs to CMVECs [7]. Thus, the elucidation of the crosstalk between CMs and CMVECs may open completely new avenues for protecting CMVECs from oxidative injury.

Exosomes, as one of cell-derived vesicles, are involved in cell-to-cell signaling and may influence processes in target cells because they can merge with and then release their contents into target cells [8]. In recent years, a large number of studies have shown the role of exosomes in different cell types and different stress conditions, such as glucose starvation [9], inflammation [10, 11], and hypoxic/ischemic preconditioning [12, 13], and indicated that exosomes induce completely different outcomes in recipient cells. Similar to many other types of cells, CMs can release exosomes, and changes in the roles of these vesicles have been related to changes in pathophysiological conditions [14, 15]. Recently, exosomes were found to be released from CMs obtained under ischemic conditions and to promote angiogenesis [16]. Hypoxic preconditioning (HPC) is widely used to simulate in vivo ischemic preconditioning (IPC) in cell culture models. HPC may enhance cellular tolerance to ROS [17]. As shown in our previous studies, miR-214, also known as “exosomal shuttle RNA,” is shuttled between cells following HPC and regulates apoptosis in target cells [13, 18]. Circular RNAs (circRNAs) are enriched and stable in exosomes [19] and can be transferred into target cells [20, 21]. However, the functions of exosomal circRNAs remain to be elucidated.

circRNAs are a novel class of noncoding RNAs that are characterized by covalently closed loop structures with neither 5′ to 3′ polarity nor a polyadenylated tail. circRNAs are expressed in a tissue-specific and developmental stage-specific manner [22]. Emerging evidence shows that circRNAs are implicated in a wide range of physiological and pathological processes, such as cell survival, growth, differentiation, and metastasis. circRNAs also regulate gene expression by acting as miRNA sponges, RNA-binding protein sequestering agents, or nuclear transcriptional regulators [23]. Several lines of evidence indicate that circRNAs are aberrantly expressed in several vascular diseases and cancers [20, 23]. For example, circRNA-MYLK can regulate the VEGFA/VEGFR2 axis by sponging miR-29a and plays a critical role in the progression of bladder carcinoma [24]. As a particularly abundant circRNA [25], circHIPK3 has been verified to be involved in regulating apoptosis, proliferation, migration, and angiogenesis by sponging different miRNAs [26–29]. However, whether circHIPK3 expression in cardiomyocyte exosomes (CMs-exos) is sensitive to hypoxic stimulation and whether circHIPK3 can be shuttled from CMs to CMVECs by exosomes remains unknown. Additionally, the regulatory roles of exosomal circHIPK3 that act as “miRNA sponges” in CMVECs are not yet elucidated. In the present study, we demonstrated that circHIPK3 can be shuttled by exosomes released from CMs pretreated with hypoxia and regulated the H_2_O_2_-induced dysfunction of CMVECs in vitro.

## 2. Materials and Methods

### 2.1. Animals

C57BL/6J mice (male and female, approximately 3 weeks old, 22-25 g) were provided and fed at Zunyi Medical University (Zunyi, China). All mice were kept in an air-conditioned room with a constant temperature of 21-22°C and had access to food and water in separated clean cages under a 12-hour light/dark cycle. All experimental procedures were performed according to the “Guide for the Care and Use of Laboratory Animals” in China and were approved by the local Experimental Animal Care and Use Committee.

### 2.2. CM Culture and Hypoxia Preconditioning

Primary cultures of neonatal mouse CMs were prepared as previously described [30] with minor modifications. Briefly, 1- to 2-day-old mice were euthanized after performing heparinization for 5-10 min. The hearts were carefully excised after quickly removing the connective tissue and atria. The ventricles were minced by eye scissors and digested with phosphate-buffered saline (PBS) containing 0.03% trypsin and 0.04% collagenase type II (Sigma) until the tissue fragments disappeared. Subsequently, a differential attachment technique was adopted to purify neonatal mouse CMs by removing cardiac fibroblasts. The resulting fraction was resuspended in a complete medium containing 10% Dulbecco's modified Eagle's medium (DMEM; Gibco) with fetal bovine serum (FBS), 1% L-glutamine, 0.1 mmol/L 5-Brdu, 1% sodium pyruvate, and 1% penicillin-streptomycin and placed in a culture flask for 90 min at 37°C. Afterwards, the cardiac fibroblasts were attached to the dishes, and the CMs remained suspended in the medium. The CMs were then seeded at a density of 1 × 10^6^ cells per well in culture flasks at 37°C in the presence of 20% O_2_, 5% CO_2_, and 75% N_2_. The CMs were then stained with cardiac troponins T (cTnT) together with 1 mg/ml 4′,6-diamidino-2-phenylindole (DAPI) (Invitrogen). Then, fluorescence microscopy (Olympus) was used to observe those cells.

The cells were stimulated with hypoxia [31]. Approximately 5 × 10^6^ CMs were separately incubated in complete media (DMEM) with 10% FBS under a 94% N_2_, 5% CO_2_, and 1% O_2_ gas mixture in a Galaxy® 48 R incubator (Eppendorf/Galaxy Corporation, USA) at 37°C for 0 h, 6 h, 12 h, or 24 h. CM viability was analyzed with CCK-8 assays according to the manufacturer's instructions.

### 2.3. CMVEC Culture and Establishment of the H_2_O_2_ Oxidative Stress Model

The isolation of CMVECs was performed according to a published protocol [32]. Briefly, C57BL/6J mice were sacrificed, and the hearts were dissected into ≈1 mm^3^ pieces. The heart samples were then digested by the addition of 0.1% collagenase II (Sigma) until the tissue blocks disappeared (approximately 30 min), followed by 0.25% trypsin-EDTA (Sigma) for 10 min at 37°C. Subsequently, the CMVECs were collected by filtrating and centrifugation (1000 xg for 10 min), resuspended in 20% FBS (Gibco)-M199 medium (HyClone) containing 50 *μ*g/ml heparin and 75 *μ*g/ml endothelial cell growth supplement (BD Biosciences), and plated in a culture flask. After 48 h, the CMVECs were cultured in a complete medium containing 20% FBS. Microscopy and flow cytometry (FCM) were adopted to identify CMVECs. Cells were incubated with the following fluorochrome-conjugated primary antibodies: anti-CD31-FITC, anti-CD34-FITC, and anti-vWF-FITC (BioLegend). CMVECs between 3 and 5 passages were used for subsequent experiments. These CMVECs were exposed to 200 *μ*M H_2_O_2_ for 3 h to establish the oxidative stress conditions for subsequent experiments [33].

### 2.4. Purification and Identification of CMs-Exosomes

The CMs exosome extraction procedures were performed as previously described [18]. Briefly, 50 ml of conditioned culture medium containing 10% exosome-depleted FBS/DMEM was used to culture CMs, and the CMs were subjected to normoxic or hypoxic conditions for 12 h. Then, the media were collected for sequential centrifugation (Optima XPN-100 ultracentrifuge; Beckman Coulter SW 41 Ti rotor) at 10,000 g for 35 min to remove cell debris, dead cells, and microvesicles. The supernatant was centrifuged at 100,000 xg for 70 min with an ultracentrifuge. Then, the exosome particles were collected and resuspended in PBS to wash, followed by another ultracentrifugation at 100,000 g for 70 min. The final particles were collected and resuspended in 200 *μ*l of PBS and stored at -80°C. The amount of CMs-exos was determined by a bicinchoninic acid (BCA) protein assay kit (Pierce). These exosomes were directly observed under a transmission electron microscope (TEM, Hitachi H7500, Tokyo, Japan) and identified by Western blotting with anti-CD63, anti-CD9, and anti-Alix antibodies (Abcam). The absolute exosome size distribution was analyzed by nanoparticle tracking analysis (NTA) (NanoSight NS300 Malvern, UK). Three recordings were performed for each sample.

### 2.5. Internalization of DiI-Labeled Exosomes into CMVECs

Exosomes were internalized by target cells as previously described, which allow the contents of exosomes to be released into target cells [34]. Briefly, 1 *μ*M DiI lipophilic dye (Invitrogen) was used to label exosomes. After incubating at 37°C for 30 min, those DiI-labeled exosomes (300 *μ*g/ml) were added to the CMVEC culture medium for 24 h. The CMVECs were then stained with 1 mg/ml DAPI (Invitrogen) for 5 min. Finally, cell fluorescence was observed by using a fluorescence microscope (Olympus) to provide.

### 2.6. 5-Ethynyl-20-deoxyuridine (EdU) Incorporation Assay

A Cell-Light EdU DNA Cell Proliferation Kit (RiboBio, Guangzhou, China) was adopted to detect the proliferation of CMVECs according to the manufacturer's protocol. After incubation with 50 mM 5-ethynyl-20-deoxyuridine (EdU) for 2 h, the CMVECs were collected and fixed in 4% paraformaldehyde and stained with Apollo Dye Solution for proliferating cells. The fluorescence intensity of cells was analyzed by FCM.

### 2.7. RNA Fluorescence In Situ Hybridization (RNA-FISH)

RNA fluorescence in situ hybridization (RNA-FISH) assays were conducted using FITC- or Cy3-labeled RNA probes targeted to circHIPK3 or miR-29a. After prehybridization (1x PBS/0.5% Triton X-100), CMVECs were hybridized overnight with RNA probes in the hybridization buffer (40% formamide, 10% dextran sulfate, 1x Denhardt's solution, 4x saline-sodium citrate (SSC), 10 mM dichlorodiphenyltrichloroethane (DDT), 1 mg/ml yeast transfer RNA, and 1 mg/ml sheared salmon sperm DNA). Cell nuclei were counterstained with DAPI, and fluorescence images were acquired.

### 2.8. Dual Luciferase Reporter Assay

CMVECs were seeded into 96-well plates in triplicate at a density of 5 × 10^3^ cells per well 24 h before transfection. The circHIPK3 sequences containing wild-type miR-29a binding sites were synthesized and inserted into the pmirGLO luciferase vector (GeneCreate, Wuhan, China), and an empty vector was used as a control. The cells were cotransfected with a mixture of firefly luciferase reporter vector, pRL-TK vector (Renilla luciferase control reporter vector) (Promega), and miR-29a mimics or negative control by using Lipofectamine 2000. In addition, 200 ng of pmirGLO-IGF-1-WT or pmirGLO- IGF-1-Mut reporter plasmids was cotransfected into cells with 50 nM miR-29a mimics using Lipofectamine 2000. Cells were harvested 48 h after transfection, and luciferase reporter assays were performed using a dual luciferase reporter assay system (Promega, Madison, WI) according to the manufacturer's instructions. Relative luciferase activity was normalized to the Renilla luciferase internal control.

### 2.9. RNA Immunoprecipitation (RIP)

CMVECs were washed twice in ice-cold PBS. Approximately 1 × 10^7^ cells were collected and resuspended in an equal volume of RNA immunoprecipitation (RIP) lysis buffer (approximately 100 ml) plus protease and RNase inhibitors. The supernatant was collected after centrifugation (10000 xg for 5 min at 4°C, ×2), and the cell lysates were incubated with 1800 *μ*l of RIP buffer (containing protease and RNA enzyme inhibitors). Magnetic beads conjugated with mouse anti-Argonaute2 (AGO2) antibody (Millipore, Billerica, MA, USA) or negative control mouse IgG (Millipore, Billerica, MA, USA) were also added to the cell lysates with rotation at 4°C overnight. Samples were washed with 500 *μ*l of RIP Wash Buffer 1 with rotation at 4°C for 15 min, followed by another wash with 500 *μ*l of RIP Wash Buffer 2 (both containing RNA enzyme inhibitors and protease inhibitors). The supernatant was collected, and 200 *μ*l of freshly NaHCO_3_ was added with rotation at 4°C for 15 min twice. The supernatant was collected, and the RNA was extracted using TRIzol and analyzed by RT-PCR or qRT-PCR to identify the presence of circRNA-HIPK3 and miR-29a.

### 2.10. Cell Transfection

CMs or CMVECs were transfected at approximately 80% confluence using a lentiviral construct (HANBIO, China) according to the manufacturer's protocol. Some of those lentiviral constructs were empty (LV), and some carried synthesized circHIPK3 (LV-circHIPK3), small interfering RNAs (Sigma) targeting circHIPK3 (LV-sicircHIPK3), or linear HIPK3 (LV-silineHIPK3). In addition, an miR-29a inhibitor, miR-29a mimics, and negative controls were all synthesized by RiboBio (Guangzhou, China) and transfected into CMVECs by using Lipofectamine 2000 (Invitrogen, USA) according to the manufacturer's instructions. CMVEC functions and gene expression were evaluated 48 h after transfection. The sequence information is listed in Table 1.

### 2.11. RNase R Treatment

Actinomycin D (2 mg/ml) or DMSO (Sigma-Aldrich, St. Louis, MO, USA) were added to the culture medium to assess the stability of circHIPK3 and its linear isoform. For RNase R treatment, approximately 2 *μ*g of total RNA was incubated with or without 3 *μ*g RNase R (Epicenter Technologies, USA) at 37°C for 30 min. The resulting RNAs were subsequently purified using an RNeasy MinElute Cleaning Kit (Qiagen), and the expression levels of circHIPK3 were determined by qRT-PCR.

### 2.12. Real-Time qPCR

Total RNA was isolated from cell lysates using TRIzol reagent (Life Technologies, Carlsbad, CA). The cDNAs of mRNA and circRNA were synthesized by using PrimeScript RT Master Mix (Takara, Dalian, China) from 500 ng of RNA. Real-time PCR analyses were performed using SYBR Premix Ex Taq II (Takara). In particular, the divergent primers annealing to the distal ends of circRNA were used to determine the abundance of circRNA. For the absolute quantification of circHIPK3, the purified PCR product amplified from the cDNA corresponding to the circHIPK3 sequence was serially diluted to generate a standard curve. Stem-loop RT-qPCR TaqMan MicroRNA assays (Life Technologies) were used to detect the amount of miRNA. GAPDH or U6 was detected as the internal reference. All primer sequences were designed and synthesized by RiboBio (Guangzhou, China). The primers are listed in Table 1. Gene expression was quantified using the 2^−*ΔΔ*Ct^ method.

### 2.13. Flow Cytometric Analysis

The apoptosis of CMVECs was determined by FCM using Annexin V-FITC/propidium iodide (PI) staining assay kits (Solarbio, China) according to the manufacturer's instructions, as previously reported [1]. CMVEC apoptosis was analyzed via a FACSCalibur flow cytometer (BD Biosciences, USA). The results are expressed as the percentage of apoptotic cells among all the cells.

Intracellular ROS production was determined by dihydroethidium (DHE) staining (Sigma, USA), followed by FCM [35] according to the manufacturer's instructions. Briefly, cells were incubated with MitoSOX reagent (2.5 mmol/l, Invitrogen) for 30 min at 37°C, washed twice with PBS, trypsinized, and centrifuged. The cell fluorescence intensity was analyzed by FCM.

### 2.14. Assessment of Intracellular SOD and MDA Levels In Vitro

CMVECs were harvested by centrifugation, and the supernatants were removed. The remaining cells were washed with PBS twice and lysed in lysis buffer for 30 min at 4°C. Following centrifugation, the supernatant was used to detect the activity of superoxide dismutase (SOD) and the level of malondialdehyde (MDA) by using different assay kits (Nanjing Jiancheng Bioengineering Institute, China), as mentioned in the protocols. The BCA test was used to quantify proteins.

### 2.15. The Terminal Deoxynucleotidyl Transferase-Mediated dUTPbiotin Nick End Labeling (TUNEL) Assay

CMVECs were previously seeded on poly-l-lysine-treated coverslips. Fixed cells were stained using a terminal deoxynucleotidyl transferase-mediated dUTPbiotin nick end labeling (TUNEL) kit according to the manufacturer's instructions (Click-iT Plus TUNEL Kit, Thermo Fisher), and cell nuclei were stained with DAPI, as previously reported [18]. For quantification, the TUNEL-positive cells were counted in at least five randomly chosen visual fields in three independent samples under a fluorescence microscope (Olympus). The total number of cells was counted using DAPI staining, and the average ratio of TUNEL-positive cells was calculated.

### 2.16. Western Blot Analysis

Western blot analysis of total protein from CMVECs was performed as previously described [29]. The protein extracts were separated by SDS-polyacrylamide gel electrophoresis (SDS-PAGE) and transferred onto polyvinylidene difluoride (PVDF) membranes. After blocking overnight in a nonfat milk solution, the membranes were probed with primary antibodies against proteins such as IGF-1, Bcl-2, Bax, procaspase-3, cleaved caspase-3, *β*-actin, or GAPDH. The PVDF membranes were incubated with horseradish peroxidase-conjugated secondary antibodies for 1 h, followed by incubation with enhanced chemiluminescence reagent (Amersham Biosciences, USA). Immunoreactivity was visualized by a ChemiDoc MP system.

### 2.17. Statistical Analysis

The data were processed with the SPSS 21.0 statistical package (IBM, Armonk, NY, USA). Measurement data were normally distributed, and the results are expressed as the mean ± standard deviation. Multiple groups were compared using one-way analysis of variance (ANOVA), followed by least significant difference (LSD) or Dunnett's T3 post hoc test for multiple comparisons. In addition, differences were considered significant at *P* < 0.05.

## 3. Results

### 3.1. Exosomes Derived from Hypoxia-Pretreated CMs Are Transferred to CMVECs and Protect CMVECs from Oxidative Damage

**Figure 1:**
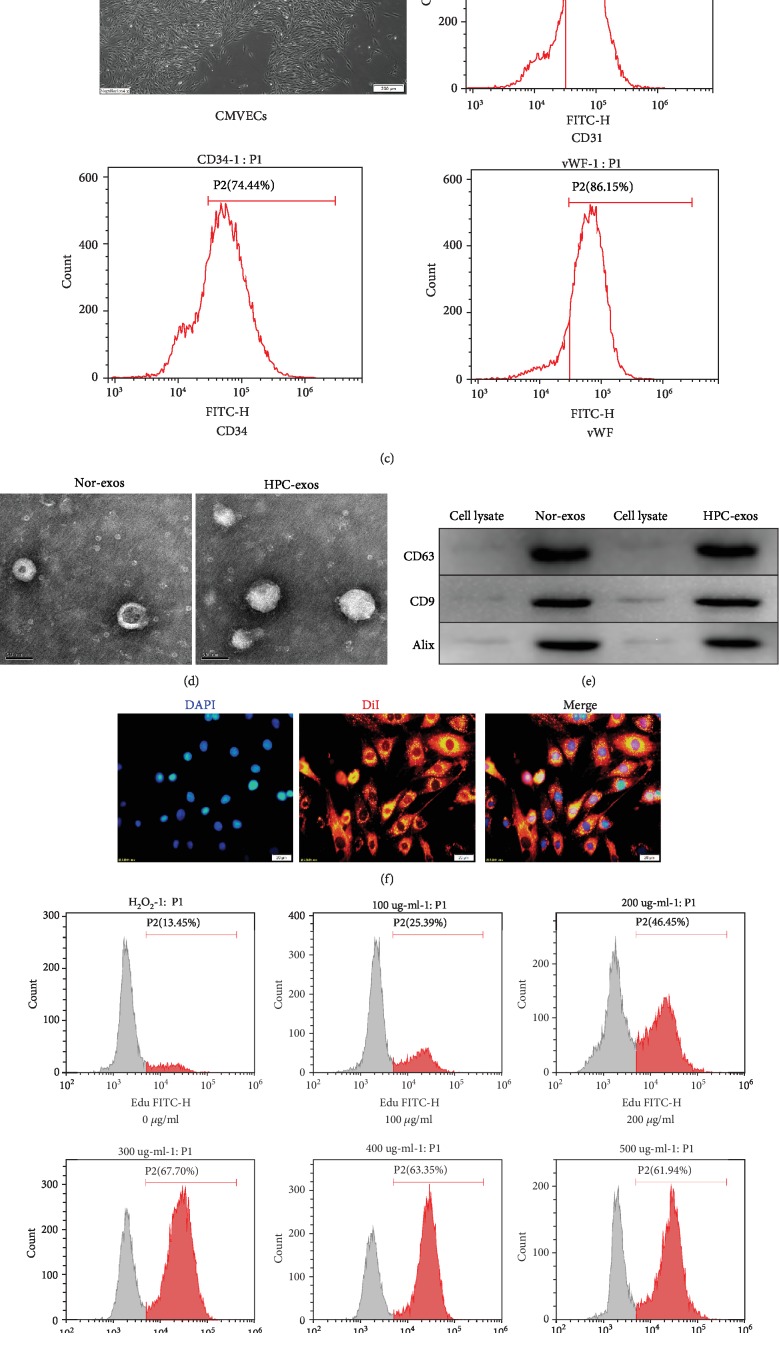
Characterization of CMs, CMVECs, exosomes, and cellular internalization. (a) Purified CMs were double stained for cTnT (red) and DAPI (blue) and observed under a fluorescence microscope (Olympus, Japan). (b) The CCK-8 assay showed that hypoxia pretreatment for 12 h significantly increased cell viability. (c) A confluent endothelial monolayer with cobblestone morphology was observed by inverted microscopy. The typical surface antigens of CMVECs, CD31, CD34, and vWF were detected by FMC. (d) Transmission electron microscopy analysis of CM-exos in two groups. Scale bar = 100 nm. (e) Western blotting of the exosome markers CD63, CD9, and Alix. (f) Fluorescence photomicrographs showing internalized DiI-labeled CM-exos (red) in DAPI-labeled CMVECs (blue). Scale bar = 20 *μ*m. (g) Representative dot plots of cell proliferation after EdU staining. (h) Quantitative analysis of proliferative cells. *n* = 3; ^∗^*P* < 0.05 compared with 0 *μ*g/ml; #*P* < 0.05 compared with 200 *μ*g/ml.

CMs were obtained [30] by using previously published methods. Immunofluorescence staining showed that the CMs expressed cTnT (Figure 1(a)). Next, we used a CCK-8 assay to examine the response of the CMs to hypoxia for 0, 6, 12, or 24 h. The cell viability remained >90% after hypoxia for 6 h and was not significantly different between normoxic and hypoxic conditions, while hypoxia for 12 h significantly increased cell viability (*P* < 0.05). However, the cell viability was significantly reduced (*P* < 0.05) after hypoxia for 24 h compared with normoxic conditions (Figure 1(b)). Therefore, we adopted the 12 h hypoxia pretreatment as the optimized treatment time.

The characteristic cobblestone morphology of CMVECs was observed under a light microscope (Figure 1(c)). FCM was used to analyze CMVECs for blood cell and endothelial markers (CD31, CD34, and vWF) [36]. The results indicated that CMVECs were positive for CD31, CD34, and vWF (Figure 1(c)).

Exosomes were isolated from conditioned media by ultracentrifugation after CMs were cultured under hypoxic or normoxic conditions. To evaluate whether hypoxia modulates the profile of exosomes released by CMs, we used TEM and NTA to assess the number, size, and morphology of the exosomes. TEM showed that the exosomes exhibited a round morphology with a cup-like shape and were approximately 30–160 nm in diameter, and the profiles of the exosomes secreted from the two groups were similar (Figure 1(d)). NTA analysis confirmed the purity of particles was approximately 90% and suggested a physically homogeneous population; the mean hydrodynamic diameter for both normoxic exosomes (Nor-exos) and hypoxic exosomes (HPC-exos) was 151 nm. The presence of the surface markers CD63, CD9, and Alix typically enriched in exosomes was detected by Western blotting in CM-exos (Figure 1(e)), which indicated that the isolation procedure gave rise to a population of vesicles highly enriched in exosomes even after the CMs were exposed to hypoxia.

Exosome internalization is one of the mechanisms for cargo delivery to recipient cells [34]. In this study, we labeled exosomes with DiI and evaluated the internalization of exosomes into CMVECs. After the labeled CM-exos (300 *μ*g/ml) were incubated with CMVECs for 24 h and counterstained with DAPI to visualize the nuclei, the fluorescence image showed that red fluorescence emission localized in the cytoplasm of the CMVECs, which indicated successful internalization of DiI-labeled exosomes by CMVECs (Figure 1(f)). Additionally, the quantification of cell proliferation with different concentrations of exosomes incubated under oxidative conditions showed that treatment of CMVECs with 300 *μ*g/ml HPC-exos 24 h could significantly improve cell proliferation. Above 300 *μ*g/ml, the cell viability did not significantly increase with increasing exosome concentration (Figures 1(g) and 1(h)). Therefore, we adopted the 300 *μ*g/ml CM-exos treatment for 24 h as the exosome treatment condition in the subsequent experiments.

**Figure 2:**
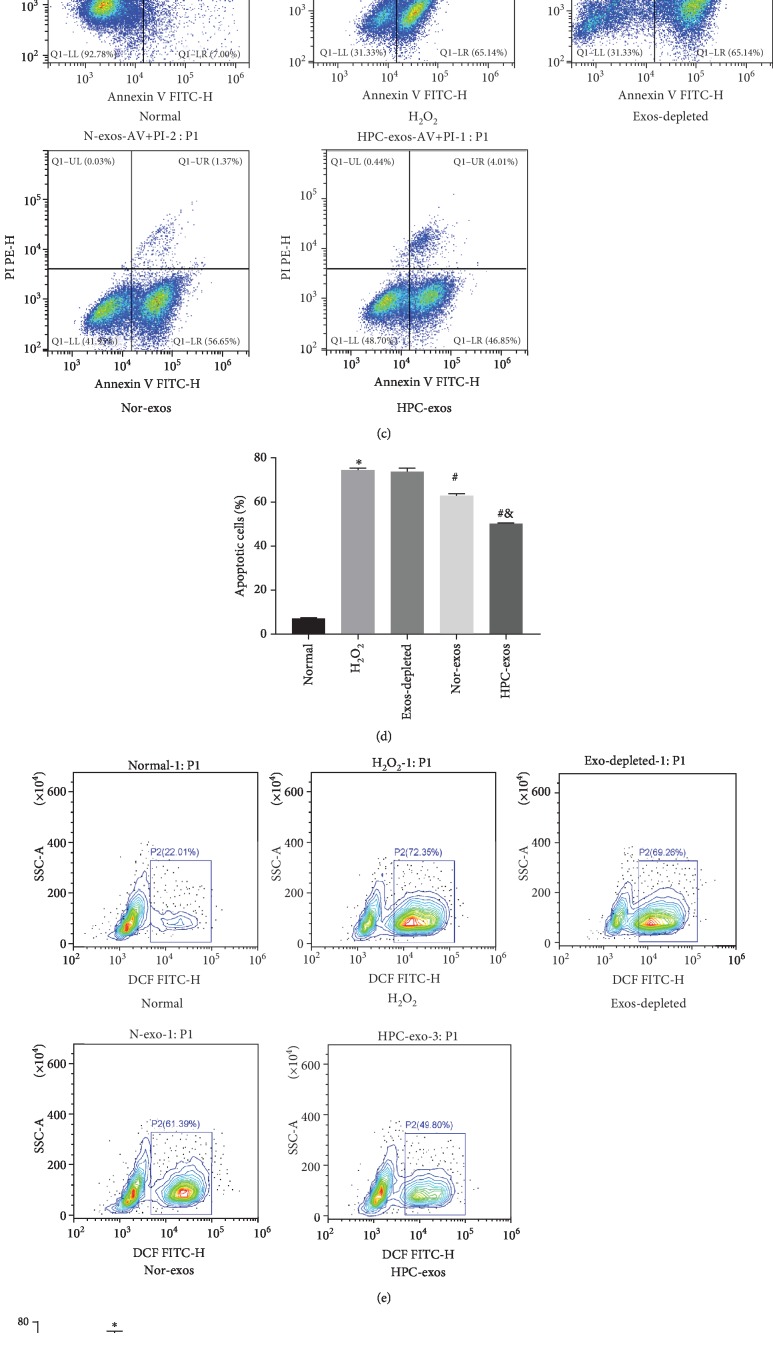
Exosomes released from hypoxia-pretreated CMs protect CMVECs from oxidative stress injury. CMVECs were pretreated with CM-exos (300 *μ*g/ml) for 24 h before incubation with 200 *μ*M H_2_O_2_ for 3 h and then subjected to analysis. (a) Representative dot plots of cell apoptosis after Annexin V/PI dual staining are shown. The left upper quadrant (% gated) shows necrotic cells (Annexin V−/PI+); the upper right quadrant (% gated) shows late apoptotic cells (Annexin V+/PI+); the lower left quadrant (% gated) shows live cells (Annexin V−/PI−); and the lower right quadrant (% gated) shows early apoptotic cells (Annexin V+/PI−). (b) The percentage of apoptotic cells represents total apoptotic cells, including both early and late apoptotic cells; *n* = 3. (c) Representative dot plots of cell apoptosis after Annexin V/PI dual staining are shown. (d) The percentage of apoptotic cells represents both early and late apoptotic cells; *n* = 3. (e) The intracellular ROS level was determined by FCM. The P2 percentage indicates the proportion of cells with increased ROS production, with signals above background 2′,7′-dichlorofluorescein (DCF) fluorescence levels. (f) Quantitative analysis of ROS levels; *n* = 3. (g) Graph represents SOD levels; *n* = 9. (h) Graph represents MDA levels; *n* = 9. (i) Representative immunofluorescence of TUNEL (green) and DAPI (blue) staining and merged images. Photos were randomly captured using a fluorescence microscope. Scale bar = 20 *μ*m. (j) The panel shows the percentage of TUNEL-positive cells; *n* = 6. (k) Apoptosis-related genes, such as procaspase-3, cleaved caspase-3, Bax, and Bcl-2, were detected by immunoblotting. (l) Quantitative analysis of the apoptosis-related proteins; *n* = 3. ^∗^*P* < 0.05 compared with the control group; ^#^*P* < 0.05 compared with the H_2_O_2_ group; ^&^*P* < 0.05 compared with the Nor-exos group.

To establish an in vitro model of CMVEC apoptosis, H_2_O_2_ (50, 100, 200, and 300 *μ*M) was selected to stimulate CMVECs for 3 h. The FCM results indicated that after 3 h of incubation, H_2_O_2_ could induce cell apoptosis in a concentration-dependent manner (*P* < 0.05). Exposure to 200 *μ*M H_2_O_2_ for 3 h resulted in the apoptosis of 72.49% of CMVECs, which was significantly higher (*P* < 0.05) than the apoptosis observed under control conditions (Figures 2(a) and 2(b)), and most of the detected apoptosis events were early apoptotic cells. Therefore, we chose 200 *μ*M H_2_O_2_ for 3 h to induce apoptosis in subsequent experiments.

To investigate the regulatory effects of CM-exos on CMVECs under oxidative stress, we evaluated the impact of Nor-exos and HPC-exos on the capacity of cells to respond to oxidative stress. CMVECs (>1 × 10^9^) were cultured with Nor-exos or HPC-exos (300 *μ*g/ml) for 24 h and then exposed to H_2_O_2_ (200 *μ*M) for 3 h to induce oxidative stress. The FCM results indicated significantly higher apoptosis rates and ROS production levels in the H_2_O_2_-treated group than in the control group. The CMVECs pretreated with CM-exos exhibited a significantly decreased percentage of apoptotic cells and reduced ROS production. Moreover, compared with Nor-exos pretreatment, HPC-exos pretreatment significantly improved the viability of CMVECs subjected to oxidative stress, suggesting that HPC-exos can be more protective than Nor-exos (Figures 2(c)–2(f)). Intracellular MDA and SOD levels, which reflect oxidation levels, were also detected by an assay kit. As shown in Figures 2(g) and 2(h), compared with the H_2_O_2_ group and the Nor-exos group, the HPC-exos group had decreased MDA levels and increased SOD activity. Next, we examined whether exosomes protected CMVECs against H_2_O_2_-induced DNA fragmentation. As shown in Figures 2(i) and 2(j), the percentage of TUNEL-positive cells was significantly increased in the H_2_O_2_-treated group compared with the control group. Furthermore, the percentage of TUNEL-positive cells was significantly reduced in the Nor-exos-treated group and the HPC-exos-treated group, compared with the H_2_O_2_ group. Moreover, HPC-exos induced more regulatory effects than the Nor-exos group. The levels of cell apoptosis-related genes, such as procaspase-3, cleaved caspase-3, Bax, and Bcl-2, were also detected by Western blotting. Not surprisingly, compared with H_2_O_2_-treated cells, HPC-exos-treated cells displayed substantially decreased levels of cleaved caspase-3 and Bax and increased levels of Bcl-2 (Figures 2(k) and 2(l)). Collectively, these results indicate that HPC-exos might exert a strong protective effect against H_2_O_2_-induced oxidative damage in CMVECs.

### 3.2. Exosomal circHIPK3 Derived from Hypoxia-Pretreated-CMs Induces the Protection of Oxidative Injury in CMVECs

**Figure 3:**
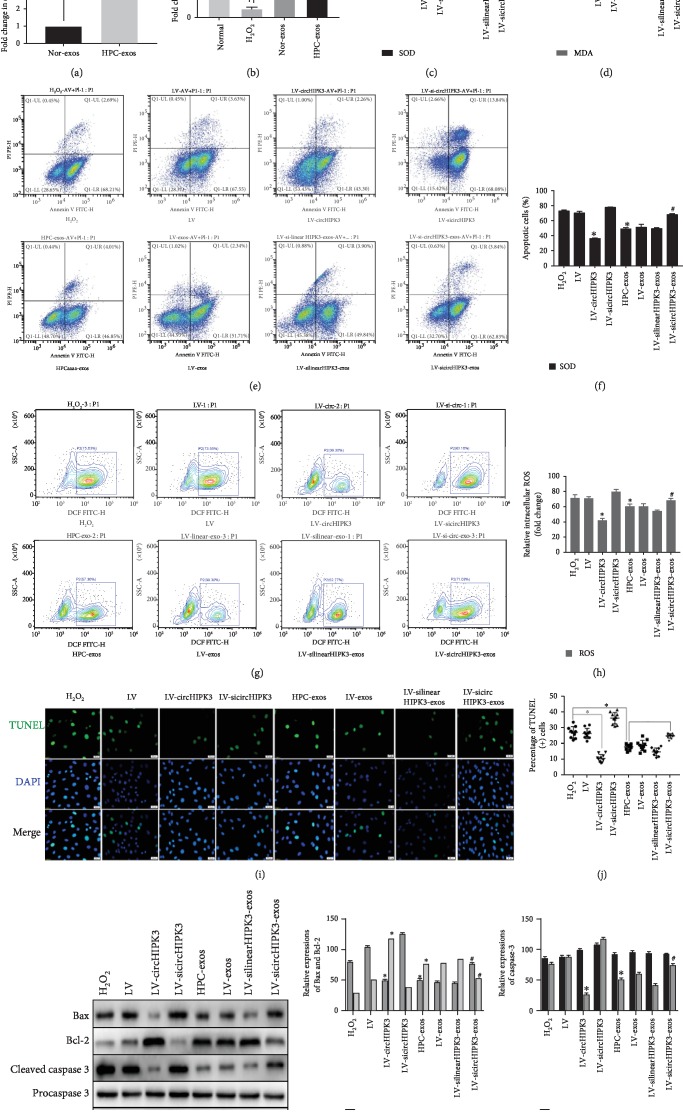
Exosomes derived from circHIPK3-enriched CMs exert an antioxidant effect on CMVECs under oxidative stress. CMVECs were transfected with negative control, circHIPK3, or circHIPK3 siRNA for 48 h. Exosomes were isolated from CMs transfected with negative control siRNA, linear circHIPK3 siRNA, and circHIPK3 siRNA and then incubated under hypoxic conditions. These exosomes were cocultured with CMVECs for 24 h and then processed under oxidative stress conditions for 2 h. (a) qRT-PCR analysis of circHIPK3 expression in exosomes after different treatments; *n* = 9. (b) qRT-PCR analysis of circHIPK3 expression in CMVECs after different treatments; *n* = 9. (c) Graph represents SOD levels; *n* = 9. (d) Graph represents MDA levels; *n* = 9. (e) Representative dot plots of cell apoptosis after Annexin V/PI dual staining are shown. (f) The percentage of apoptotic cells represents both early and late apoptotic cells; *n* = 3. (g) The intracellular ROS level was determined by FCM. The P2 percentage indicates the proportion of cells with increased ROS production, with signals above background 2′,7′-dichlorofluorescein (DCF) fluorescence levels. (h) Quantitative analysis of the ROS levels; *n* = 3. (i) Representative immunofluorescence of TUNEL (green) and DAPI (blue) staining and merged images. Photos were randomly captured using a fluorescence microscope. Scale bar = 20 *μ*m. (j) The panel shows the percentage of TUNEL-positive cells; *n* = 6. (k) Apoptosis-related genes, such as procaspase-3, cleaved caspase-3, Bax, and Bcl-2, were detected by immunoblotting. (l, m) Quantitative analysis of the apoptosis-related proteins; *n* = 3. ^∗^*P* < 0.05 compared with the H_2_O_2_ group; ^#^*P* < 0.05 compared with the HPC-exos group.

circRNA can be transferred into target cells by exosomes [21]. Exosomes derived from CMs cultured under hypoxic conditions have a greater reparative capacity than exosomes from normal cells. It is important to investigate the content of circRNA with potential biological functions in exosomes released under certain hypoxic conditions. circBase retrieval revealed that the HIPK3 host gene might produce 20 circRNAs in the human genome and 3 circRNAs in the mouse genome. One circRNA (mmu_circ_0001052) from the HIPK3 host gene in mice was identified in endothelial cells followed by RNase R treatment and could regulate endothelial proliferation and vascular dysfunction [29]. qRT-PCR assays revealed that circHIPK3 was significantly upregulated in HPC-exos compared with Nor-exos (Figure 3(a)). circHIPK3 expression was also detected in CMVECs after pretreatment with HPC-exos or Nor-exos (Figure 3(b)). qRT-PCR analysis revealed that circHIPK3 levels were substantially downregulated in CMVECs treated with H_2_O_2_ compared with control CMVECs. Compared with Nor-exos pretreatment, HPC-exos pretreatment significantly rescued the circHIPK3 levels of CMVECs subjected to oxidative stress, suggesting the existence of a possible negative connection between circHIPK3 and H_2_O_2_-induced oxidative damage in CMVECs.

We next investigated the role of circHIPK3 in CMVECs. First, we conducted gain-of-function and loss-of-function analyses of circHIPK3 and determined whether circHIPK3 alone was sufficient to protect CMVECs from oxidative damage. Cells were transfected with the circHIPK3 expression lentiviral vector (LV-circHIPK3), siRNA-circHIPK3 expression lentiviral vector (LV-sicircHIPK3), or lentiviral vector empty vector (LV). In CMVECs under oxidative conditions, circHIPK3 overexpression significantly decreased apoptosis and oxidative status (including ROS, SOD, and MDA) (Figures 3(c)–3(j)), upregulated the antiapoptotic protein Bcl-2, and downregulated the proapoptotic proteins Bax and cleaved caspase-3 (Figures 3(k)–3(m)). Additionally, siRNAs were designed for circHIPK3 silencing (LV-sicircHIPK3), which was verified to significantly downregulate circHIPK3 expression [29]. FCM analysis and TUNEL assays revealed that compared with H_2_O_2_ treatment, siRNA transfection did not markedly affect CMVECs apoptosis or ROS production (Figures 3(c)–3(h)). LV-sicircHIPK3 clearly increased the levels of MDA and the proapoptotic proteins Bax and cleaved caspase-3 and decreased SOD production and the antiapoptotic protein Bcl-2; the LV-sicircHIPK3 and H_2_O_2_ treatment groups were not significantly different (Figures 3(i)–3(k)).

**Figure 4:**
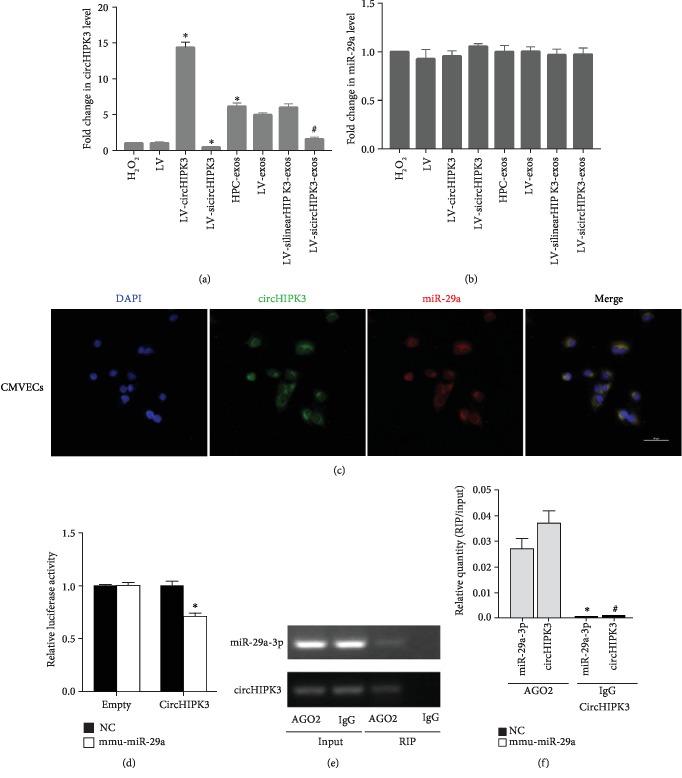
circHIPK3 directly binds to miR-29a. (a) qRT-PCR analysis of the expression of circHIPK3 in CMVECs after different treatments; *n* = 9; ^∗^*P* < 0.05 compared with the H_2_O_2_ group; ^∗^*P* < 0.05 compared with the HPC-exos group. (b) qRT-PCR analysis of the expression of miR-29a in CMVECs after different treatments. (c) FISH for circHIPK3 (green) and miR-29a (red) was detected in CMVECs. Scale bar = 50 *μ*m. (d) The relative luciferase activities were analyzed by cotransfection with miR-29a mimics or miR-NC and luciferase reporter vectors pmirGLO-circHIPK3-WT or pmirGLO-circHIPK3-empty. *n* = 3, ^∗^*P* < 0.05 compared with the empty group. (e, f) Anti-AGO2 RIP was performed in CMVECs transfected with miR-29a mimics or circHIPK3, followed by RT-PCR and qRT-PCR to detect miR-29a and circHIPK3. *n* = 9; ^∗^*P* < 0.05 compared with the miR-29a-3p-AGO2 group. ^#^*P* < 0.05 compared with the circHIPK3-AGO2 group.

To further determine whether the effects of HPC-exos on CMVECs are dependent on circHI-PK3 and not linear HIPK3, the impact of linearHIPK3 or circHIPK3 loss-of-function in HPC-exos was determined. CMs were pretreated with negative control siRNA, linear HIPK3 siRNA, or circHIPK3 siRNA for 48 h. After transfection, the cells were incubated under hypoxic conditions for 12 h, and their exosomes (named LV-exos, LV-silinearHIPK3-exos, and LV-sicircHIPK3-exos) were collected. Then, the exosomes were cocultured with CMVECs. Interestingly, LV-sicircHIPK3-exos but not LV-exos or LV-silinearHIPK3-exos could partially neutralize the protective effect of HPC-exos; compared with the HPC-exos group, the LV-sicircHIPK3-exos group displayed increased apoptosis and ROS production (Figures 3(c)–3(m)). qRT-PCR analysis of circHIPK3 expression revealed that compared with CMVECs treated with H_2_O_2_, CMVECs pretreated with HPC-exos or transfected with circHIPK3 had significantly increased circHIPK3 levels, whereas circHIPK3 siRNA-pretreated CMVECs displayed a further decrease in circHIPK3 expression under oxidative stress. Interestingly, compared with the HPC-exos group, CMVECs pretreated with LV-sicircHIPK3-exos had significantly decreased circHIPK3 levels. In contrast, linear HIPK3 siRNA had no effect on regulating circHIPK3 expression (Figure 4(a)). These data confirmed the antioxidant function of circHIPK3 and suggested that rescuing downregulated circHIPK3 expression in CMVECs with HPC-exos is a potential strategy for protecting CMVECs from oxidative stress injury.

### 3.3. circHIPK3 Abundantly Sponges miR-29a in CMVECs

Stable transcripts with many miRNA-binding sites may function as miRNA sponges. circHIPK3 was mainly expressed in the cytoplasm of CMVECs. A previous study demonstrated that circHIPK3 could act as a miR-29a sponge and regulate cell growth [37]. We thus investigated the regulatory relationship between miR-29 and circHIPK3 expression levels in CMVECs. qRT-PCR showed that there was no significant change in the expression of miR-29a in each group (Figure 4(b)). If circHIPK3 indeed interact with miR-29a, circHIPK3 and miR-29a should be coexpressed in CMVECs. FISH assays showed that circHIPK3 and miR-29a were colocalized in the cytoplasm (Figure 4(c)). Next, we carried out luciferase reporter assays and demonstrated that the overexpression of miR-29a significantly decreased the luciferase activity of the vector containing the complete circHIPK3 sequence but did not affect the luciferase activity of the empty vector in CMVECs (Figure 4(d)). Furthermore, the AGO2 protein is a core component of the RNA-induced silencing complex (RISC) that binds miRNA complexes to target mRNAs or circRNAs. We conducted anti-AGO2 RIP in CMVECs transiently overexpressing miR-29a to pull down circHIPK3 using anti-AGO2 antibodies or control IgG, followed by RT-qPCR analysis. We observed increased enrichment of circHIPK3 in the miR-29a-captured fraction compared to the lgG fraction in RIP (Figures 4(e) and 4(f)), suggesting that miR-29a could directly target circHIPK3 in an AGO2-dependent manner. The above results implied that circHIPK3 can bind miR-29a but cannot change the expression of miR-29a.

### 3.4. miR-29a Upregulation in CMVECs Promotes Cell Apoptosis and Increases ROS Levels by Targeting IGF-1 In Vitro

**Figure 5:**
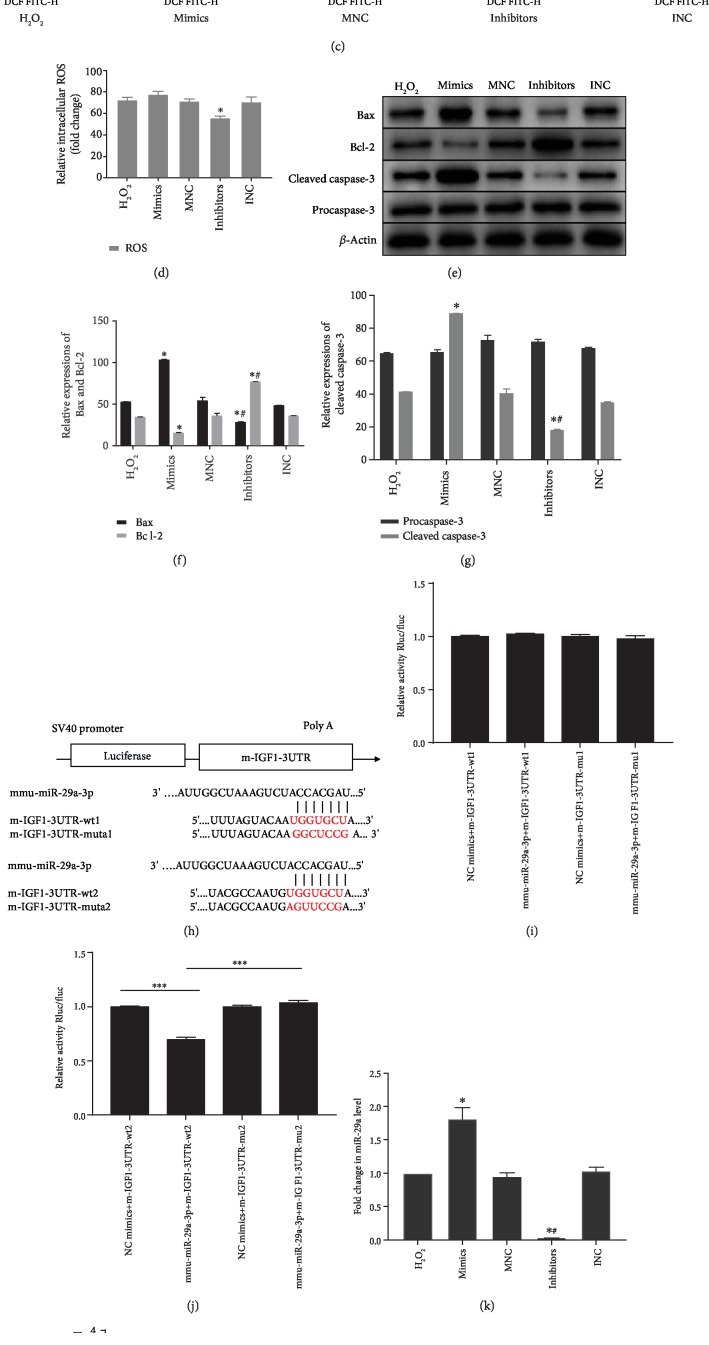
Overexpression of miR-29a promotes apoptosis through targeting IGF-1 in CMVECs. (a) Representative dot plots of cell apoptosis after Annexin V/PI dual staining are shown. (b) The percentage of apoptotic cells represents both early and late apoptotic cells; *n* = 3. (c) The intracellular ROS level was determined by FCM. The P2 percentage indicates the proportion of cells with increased ROS production, with signals above the background 2′,7′-dichlorofluorescein (DCF) fluorescence levels. (d) Quantitative analysis of the ROS levels; *n* = 3. (e) Apoptosis-related genes, such as procaspase-3, cleaved caspase-3, Bax, and Bcl-2, were detected by immunoblotting. (f, g) Quantitative analysis of the apoptosis-related proteins; *n* = 3. (h) A schematic diagram of the putative binding between IGF-1 and miR-29a. (i, j) The relative luciferase activities were analyzed by cotransfecting miR-29a mimics or miR-NC and the luciferase reporter vectors pmirGLO-LGF-1-Mut or pmirGLO-IGF-1-WT. (k) qRT-PCR analysis of miR-29a expression in CMVECs after different treatments; *n* = 9. (l) qRT-PCR analysis of IGF-1 mRNA expression in CMVECs after different treatments; *n* = 9. (m) IGF-1 protein expression was detected by immunoblotting. (n) Quantitative analysis of IGF-1 protein expression; *n* = 3. ^∗^*P* < 0.05 compared with the H_2_O_2_ group; ^#^*P* < 0.05 compared with the mimics group; ^∗∗∗^*P* < 0.05 compared with the NC-mimics-IGF1-WT group; ^∗∗∗∗^*P* < 0.05 compared with the Mmu-mimics-IGF1-WT group.

To investigate the role of miR-29a in CMVECs, miR-29a gain-of-function and loss-of-function treatments were used to evaluate apoptosis and ROS status in CMVECs. The data showed that compared with the H_2_O_2_ treatment, the overexpression of miR-29a induced apoptosis and increased ROS levels, but the difference was not statistically significant. However, the inhibition of miR-29a significantly protected CMVECs from apoptosis and oxidative stress injury following H_2_O_2_ insult (Figures 5(a)–5(d)). The levels of apoptosis-related genes were also detected by Western blotting. The proapoptotic genes cleaved caspase-3 and Bax were substantially decreased, and the antiapoptotic gene Bcl-2 was significantly increased in the inhibitor group compared with the H_2_O_2_ group (Figures 5(e)–5(l)). Subsequently, we searched the miRanda database to predict miR-29a targets. As shown in Figure 5(h), the 3′ untranslated region (3′-UTR) of IGF-1 contains two putative binding motifs of miR-29a. Increasing evidence indicates that IGF-1 is involved in the inhibition of apoptosis through cellular signal transduction and metabolic mechanisms [38, 39]. In addition, miR-29a has been shown to regulate the apoptosis of MCs by repressing the expression of IGF-1 [40]. Luciferase reporter assays were performed to verify that IGF-1 is a target of miR-29a in CMVECs. The data showed that compared to that of miR-NC, the transfection of miR-29a mimics could reduce the activity of a luciferase reporter carrying the wild-type IGF-1-3′UTR (Figures 5(i) and 5(j)). Furthermore, the miR-29a mimics significantly increased and the miR-29a inhibitor significantly decreased miR-2an expression in CMVECs (Figure 5(k)). We also found that miR-29a mimics significantly reduced IGF-1 mRNA levels, whereas the miR-29a inhibitor induced the opposite effect in CMVECs (Figure 5(l)). Western blot showed that miR-29a downregulation markedly promoted IGF-1 expression, while the upregulation of miR-29a clearly reduced IGF-1 levels (Figures 5(m) and 5(n)). These results indicated that miR-29a could target IGF-1 and negatively regulate its expression and that miR-29a could promote oxidative damage in CMVECs partially through targeting IGF-1.

### 3.5. Exosomal circHIPK3 Derived from Hypoxia-Pretreated CMs Induces Protection from Oxidative Injury via miR-29a/IGF-1 in CMVECs

**Figure 6:**
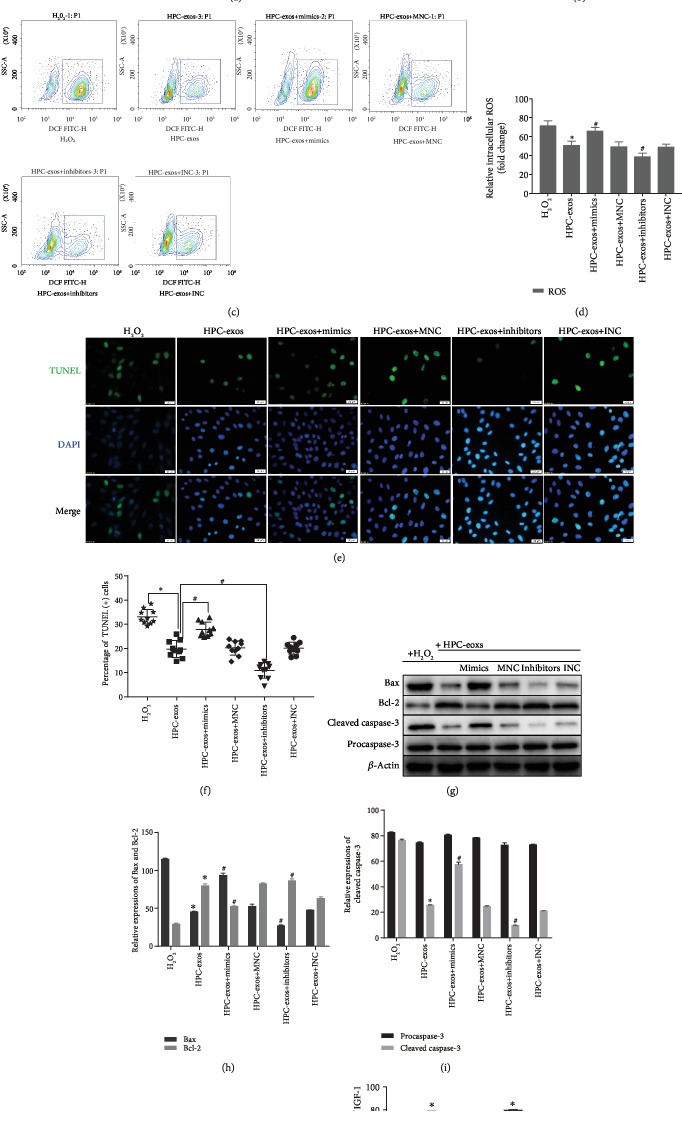
HPC-exos protect against oxidative injury via miR-29a/IGF-1 in CMVECs. (a) Representative dot plots of cell apoptosis after Annexin V/PI dual staining are shown. (b) The percentage of apoptotic cells represents both early and late apoptotic cells; *n* = 3. (c) The intracellular ROS level was determined by FCM. The P2 percentage indicates the proportion of cells with increased ROS production, with signals above the background 2′,7′-dichlorofluorescein (DCF) fluorescence levels. (d) Quantitative analysis of ROS levels; *n* = 3. (e) Representative immunofluorescence of TUNEL (green) and DAPI (blue) staining and merged images. Photos were randomly captured using a fluorescence microscope. Scale bar = 20 *μ*m. (f) The panel shows the percentage of TUNEL-positive cells; *n* = 6. (g) Apoptosis-related genes, such as procaspase-3, cleaved caspase-3, Bax, and Bcl-2, were detected by immunoblotting. (h, i) Quantitative analysis of the apoptosis-related proteins; *n* = 3. (j) IGF-1 protein expression was detected by immunoblotting. (k) Quantitative analysis of IGF-1 protein expression; *n* = 3. ^∗^*P* < 0.05 compared with the H_2_O_2_ group; ^#^*P* < 0.05 compared with the HPC-exos group.

To investigate whether HPC-exos regulated oxidative damage in CMVECs by targeting IGF-1 via miR-29a sponging, we transfected CMVECs with miR-29a mimics, inhibitor, or negative control RNA. 48 h after transfection, HPC-exos were added to CMVECs for 24 h, and then the CMVECs were exposed to oxidative stress for 3 h. Compared with the H_2_O_2_ group, the HPC-exos group displayed substantially reduced apoptosis, whereas the HPC-exos+mimics group had significantly elevated apoptosis and the HPC-exos+inhibitor group had significantly decreased apoptosis (Figures 6(a) and 6(b)). The production of ROS was measured to further confirm the antioxidant ability of HPC-exos. The ROS production of the HPC-exos+mimics group was dramatically increased compared with that of the HPC-exos group. In contrast, ROS was further decreased in the HPC-exos+inhibitor group compared with the HPC-exos group in CMVECs, and the difference was statistically significant (Figures 6(c) and 6(d)). The percentage of TUNEL-positive cells was significantly increased in the HPC-exos+mimics group compared with the HPC-exos group. Furthermore, the percentage of TUNEL-positive cells was significantly decreased in the HPC-exos+inhibitor group compared with the HPC-exos group (Figures 6(e) and 6(f)). Apoptosis-related proteins were then detected by Western blotting. Indeed, compared with the H_2_O_2_-treated cells, cells treated with HPC-exos or HPC-exos+miR-29a inhibitor displayed substantially decreased expression of the proapoptotic proteins cleaved caspase-3 and Bax and increased expression of the antiapoptotic protein Bcl-2. However, cells treated with HPC-exos+miR-29a mimics displayed the opposite results; caspase-3 and Bax levels were increased while Bcl-2 levels were decreased in the HPC-exos+mimics group compared with the H_2_O_2_ group (Figures 6(g)–6(i)). Next, IGF-1 protein levels were detected by Western blotting. In CMVECs exposed to H_2_O_2_, the IGF-1 protein levels of the HPC-exos+mimics and HPC-exos+inhibitor groups were downregulated and upregulated, respectively, compared with that of the HPC-exos group (Figures 6(j) and 6(k)). These data suggested that HPC-exos function as antioxidants to regulate CMVECs via the miR-29a/IGF-1 axis.

**Figure 7:**
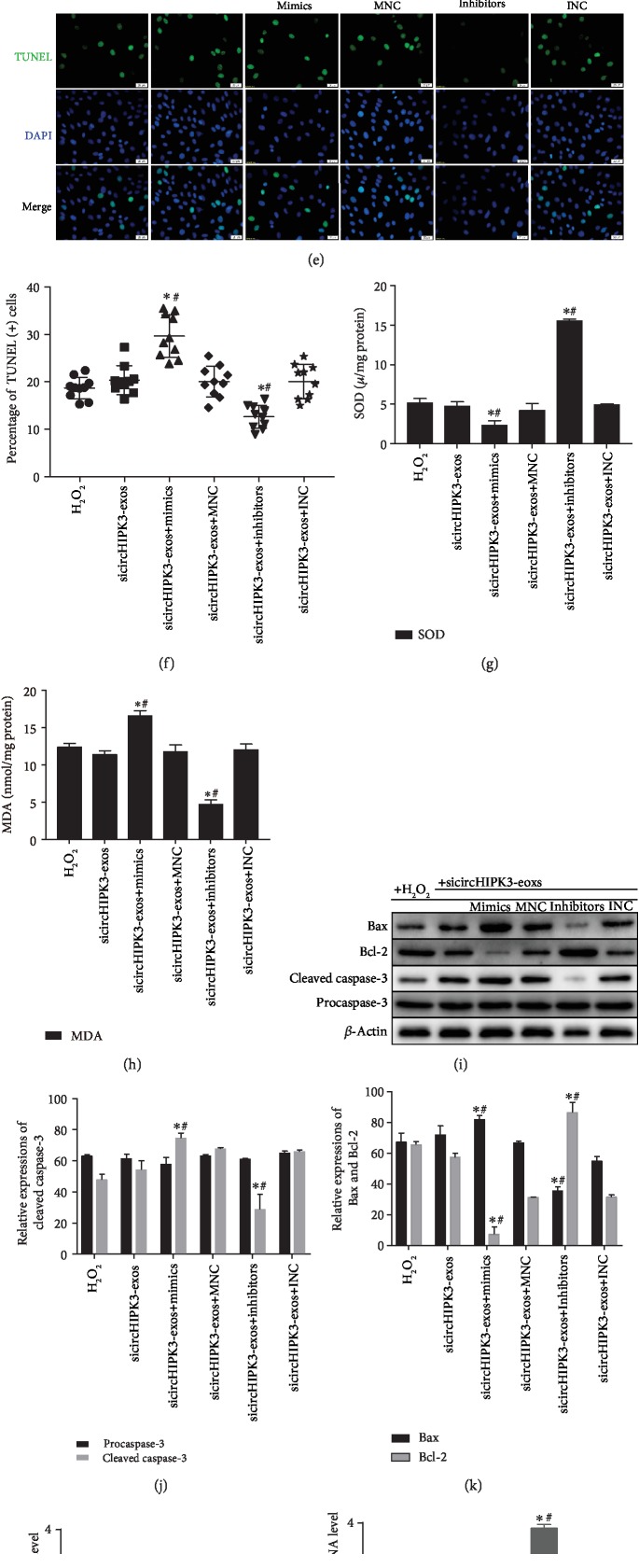
Exosomal circHIPK3 derived from hypoxia-pretreated CMs induces protection from oxidative injury via miR-29a/IGF-1 in CMVECs. (a) Representative dot plots of cell apoptosis after Annexin V/PI dual staining are shown. (b) The percentage of apoptotic cells represents both early and late apoptotic cells; *n* = 3. (c) The intracellular ROS level was determined by FCM. The P2 percentage indicates the proportion of cells with increased ROS production, with signals above background 2′,7′-dichlorofluorescein (DCF) fluorescence levels. (d) Quantitative analysis of ROS levels; *n* = 3. (e) Representative immunofluorescence staining of TUNEL (green) and DAPI (blue) staining and merged images. Photos were randomly captured using a fluorescence microscope. Scale bar = 20 *μ*m. (f) The panel shows the percentage of TUNEL-positive cells; *n* = 6. (g) Graph represents SOD levels; *n* = 9. (h) Graph represents MDA levels; *n* = 9. (i) Apoptosis-related genes, such as procaspase-3, cleaved caspase-3, Bax, and Bcl-2, were detected by immunoblotting. (j, k) Quantitative analysis of the apoptosis-related proteins; *n* = 3. (l) qRT-PCR analysis of miR-29a expression in CMVECs after different treatments. (m) qRT-PCR analysis of IGF-1 mRNA expression in CMVECs after different treatments. (n) IGF-1 protein was detected by immunoblotting. (o) Quantitative analysis of IGF-1 protein; *n* = 3. ^∗^*P* < 0.05 compared with the H_2_O_2_ group; ^#^*P* < 0.05 compared with the sicircHIPK3-exos group.

To identify whether the circHIPK3 in HPC-exos regulates CMVECs oxidative damage and IGF-1 expression by inhibiting miR-29a, we performed rescue experiments. Similar to the previous experiments in this study, CMVECs were transfected with miR-29a mimics, inhibitor, or negative control RNA. Forty-eight hours after transfection, sicircHIPK3-exos were added to CMVECs for 24 h, and then CMVECs were exposed to oxidative stress for 3 h. As shown in Figures 7(a) and 7(b), the CMVEC apoptosis induced by circHIPK3 knockdown in HPC-exos was rescued by the miR-29a inhibitor but intensified by the miR-29a mimics. Furthermore, in vitro oxidative damage (including ROS, SOD, and MDA) was increased with the combination of exosomal circHIPK3 silencing and miR-29a overexpression compared with circHIPK3 knockdown in HPC-exos alone. In addition, we also found that oxidative stress in CMVECs was suppressed with the combined knockdown of miR-29a in CMVECs and circHIPK3 in HPC-exos compared with exosomal circHIPK3 silencing alone (Figures 7(c)–7(k)). In addition, we did not observe a marked increase in miR-29a levels following circHIPK3 knockdown in HPC-exos; moreover, the miR-29a mimics significantly increased and the miR-29a inhibitor significantly decreased miR-29a expression in CMVECs (Figure 7(l)). However, the mRNA and protein levels of IGF-1 were significantly decreased in the sicircHIPK3 and sicircHIPK3+mimic groups (Figures 7(l)–7(o)). Interestingly, the suppression of IGF-1 by sicircHIPK3-exos or miR-29a mimics could also be rescued by the addition of the miR-29a inhibitor (Figures 7(l)–7(o)). These data suggested that HPC-exosomal circHIPK3 regulate oxidative damage in CMVECs via the miR-29a/IGF-1 axis.

## 4. Discussion

circRNAs have recently gained attention due to their key roles in the regulation of gene expression and human diseases. In exosomes, circRNAs are enriched and stable [19] and can be transferred into target cells [20, 21]. However, few exosomal circRNAs have been explored, especially under certain pathological conditions. In this study, we investigated the role of exosomal circHIPK3 released from CMs pretreated with hypoxia in maintaining cardiac microvascular endothelial cell function. Mechanistically, circHIPK3 in HPC-exos acts as an endogenous miR-29a sponge to inhibit miR-29a activity, thereby leading to increased IGF-1 expression and regulating oxidative damage in CMVECs in vitro.

Compared to damage to CMs, damage to microvasculature and microendothelial cells in the context of ischemic cardiomyopathy has unfortunately been a neglected topic in the past 30 years [41]. Oxidative stress-related complications in CMVECs after myocardial ischemia are the major cause of heart dysfunction [42]. Considering the central role of microcirculation in the exchange of oxygen and metabolites between blood and CMs [43], it is important to provide protection for microvasculature from oxidative injury. Recently, studies examining the intercellular interactions between the different cell types in the heart have provided new insight into the regulatory mechanism of CMVEC dysfunction. The close contact between CMs and CMVECs allows exosomes to transfer from CMs to CMVECs [12], which may represent completely new avenues for the regulation of oxidative damage in CMVECs.

Exosomes, as the beneficial paracrine signals generated by different cell types, carry functional messages and play an essential role in cell-to-cell communication under both physiological and pathophysiological conditions [44]. HPC is widely used to enhance cellular tolerance to oxidative stress [17]. Importantly, HPC enhanced the benefit of CMs-exos in an animal myocardial infarction model and led to an increased proangiogenic effect of exosomes [16]. In this study, cell viability analysis showed that 12 h was the appropriate length for hypoxia preconditioning in CMs. We obtained exosome vesicles (round, 30 ± 100 nm) from the media of conditioned CMs and confirmed these vesicles by detecting the expression of specific surface markers. The number, size, and morphology of exosomes were not changed after exposure to hypoxia. Furthermore, the exosomes could be internalized by CMVECs, which indicates that these exosomes may play roles in CMVECs by transferring cargo. H_2_O_2_ induces oxidative stress, which may cause cell damage [45]. We established an in vitro oxidative stress model with different concentrations of H_2_O_2_ to simulate the microenvironment of infarcted myocardium. Given that 200 *μ*M H_2_O_2_ induced the most apoptosis (up to 72.49%) among the tested concentrations and relatively low necrosis in CMs, we chose 200 *μ*M H_2_O_2_ pretreatment for 3 h to establish an in vitro model of oxidative stress. Furthermore, cell proliferation measurements of cells incubated with different concentrations of exosomes under hypoxic conditions showed that 300 *μ*g/ml could significantly improve the proliferation of CMVECs pretreated with H_2_O_2_. We have shown that the exosomes generated by CMs under hypoxic conditions increase the survival of CMVECs to a greater extent than exosomes from normoxic cells.

Exosomes contain specific proteins, RNA, ncRNA (including circRNA, lncRNA, and miRNA), and lipids. Recently, reports have demonstrated that the transfer of unique exosome-derived miRNAs and circRNAs to recipient cells is an alternative mechanism that allows for gene-based communication between cells, in addition to the classical mechanisms, including direct cell-cell contact or chemical receptor-mediated events [13, 20]. In recent years, circRNAs have emerged as novel regulators in the pathogenesis of several ocular diseases, including retinal vascular dysfunction in diabetes retinopathy [29] and physiological neuronal apoptosis in developing rat retina [46]. In addition, circRNA is dynamically regulated after cells are exposed to hypoxia [47]. circRNA HIPK3, originating from exon 2 of the HIPK3 gene, is highly conserved in different cell lines and tissues [26, 37]. circBase retrieval revealed that the HIPK3 host gene might produce 20 circRNAs in the human genome and 3 circRNAs in the mouse genome. One circRNA (mmu_circ_0001052) from the HIPK3 host gene in mice was identified in endothelial cells after RNase R treatment and could increase endothelial proliferation and vascular dysfunction [29]. However, another study demonstrated a contrasting role of circHIPK3; the overexpression of circHIPK3 inhibits the migration, invasion, and angiogenesis of human invasive bladder cancer T24T and UMUC3 cells [26]. Here, we found that hypoxia preconditioning significantly upregulates circHIPK3 expression in CM-exos and that H_2_O_2_ significantly downregulates circHIPK3 expression in CMVECs. We thus hypothesized that circHIPK3 downregulation is responsible for H_2_O_2_-induced CMVEC dysfunction. In addition, HPC-exos could induce circHIPK3 upregulation and protect CMVECs from apoptosis and oxidative damage, which prompted us to further investigate the function of circHIPK3. We show that the overexpression of circHIPK3 alone was sufficient to protect CMVECs from oxidative damage, whereas circHIPK3 knockdown further increased the apoptosis, ROS level, and MDA level of CMVECs, implying that decreased circHIPK3 levels in CMVECs promote the dysfunction of CMVECs under oxidative stress. Furthermore, the knockdown of circHIPK3, but not linear HIPK3, in HPC-exos increased the apoptosis and oxidative stress of CMVECs. Taken together, the circHIPK3 from HPC-exos mediates CMVEC protection.

circRNAs have emerged as promising novel regulators of gene expression through a complicated network involving mRNAs, miRNAs, and proteins [48]. A previous study showed that circHIPK3 sponged to 18 sites of 9 miRNAs (miR-124, miR-152, miR-193a, miR-29a, miR-29b, miR-338, miR-379, miR-584, and miR-654) [37]. miR-29a, a well-known angiogenesis suppressor, was proposed to participate in the process of apoptosis and proliferation in endotheliocytes [49–51]. In our experiment, we showed that the mRNA levels of miR-29a were not influenced by the overexpression or knockdown of circHIPK3. This result occurred because circRNA acts as an endogenous miRNA sponge to adsorb miRNA and inhibit miRNA activity, instead of degrading miRNA [29]. In addition, FISH assays showed that circHIPK3 and miR-29a were colocalized in the cytoplasm of CMVECs. The dual luciferase reporter assay showed that circHIPK3 binds to miR-29a. Furthermore, increased circHIPK3 expression could lead to a binding platform for AGO2 and function as a miRNA sponge for miR-29a. These results further suggest that circHIPK3 can specifically bind to miR-29a.

Canonically, miRNAs can either retard the translation or induce the degradation of target mRNA and then decrease the expression of the corresponding protein by incomplete or complete base pairing to the 3′UTR of target mRNA [52]. Many targets of miR-29a have been identified, such as VEGF in gastric carcinoma [49] and MCL1 in anaplastic large cell lymphomas [53]. We demonstrate that IGF-1, which is of vital significance in the maintenance of CMVECs, is a newly discovered direct target of miR-29a in CMVECs. The miR-29a-induced imbalance in IGF-1 expression may promote damage accumulation and attenuate the protective effect of IGF-1. We established in vitro miR-29a gain-of-function and loss-of-function models to assess the effect of miR-29a on oxidative damage. In agreement with previous studies [38], our data also indicated that the miR-29a inhibitor significantly inhibited CMVEC oxidative stress injury and alleviated apoptosis; moreover, these inhibitory effects were strikingly similar to the overexpression of circHIPK3. We also found that upregulated miR-29a levels effectively decreased IGF-1 mRNA and protein expression, while downregulated miR-29a levels significantly increased IGF-1 protein expression. In addition, a dual luciferase reporter assay showed that miR-29a binds to IGF-1. miR-29a mimics abrogated the HPC-exos-mediated repressive effects though increasing apoptosis and ROS under oxidative conditions in vitro. In contrast, transfection of the miR-29a inhibitor significantly decreased apoptosis and oxidative damage in CMVECs. Furthermore, the high level of circHIPK3 in HPC-exos is a sink for miR-29a and releases the inhibitory effect of miR-29a on IGF-1. The rescue experiments also showed that the reduced protection from oxidative damage induced by circHIPK3 knockdown in HPC-exos could be rescued by the miR-29a inhibitor and intensified by the miR-29a mimics. Furthermore, the suppression of IGF-1 by sicircHIPK3-exos could also be rescued after miR-29a inhibition. This regulatory mechanism provides a novel insight into microvascular dysfunction.

**Figure 8:**
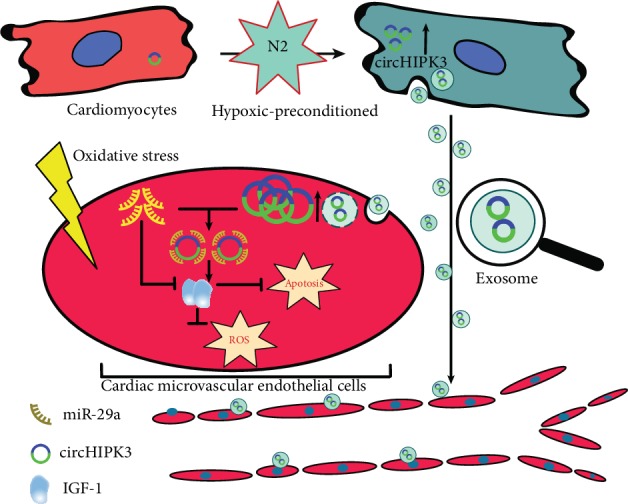
A schematic cartoon of the circHIPK3/miR-29g/IGF-1 axis in CMVECs. In CMs, circHIPK3 is upregulated by hypoxia pretreatment. Then, circHIPK3 is internalized by CMVECs through exosomes. In CMVECs, circHIPK3 sponges additional endogenous miR-29a to sequester and inhibit miR-29a activity, thereby leading to increased IGF-1 expression, which regulates oxidative damage in cardiac microvascular endothelial cells.

In conclusion, we observed circHIPK3 upregulation in HPC-exos. Our results demonstrated that the circHIPK3 in CMs-exos could influence the survival of CMVECs by sponging endogenous miR-29a to sequester and reduce miR-29a activity, thus resulting in the increased expression of the miR-29a-targeted gene IGF-1 (Figure 8). This study shed light on a potentially new targeted method to regulate CMVECs dysfunction using an exosomal ncRNA-based approach. Additional in vivo studies are warranted to further confirm that CMs-exos pretreated with hypoxia have similar effects on the survival of CMVECs. Notably, CMs-exos contain various types of circRNA, including circHIPK3; moreover, circHIPK3 targets more than one miRNA, and IGF-1 is not the only protein downstream of miR-29a. Furthermore, the upstream signaling mechanism that causes the change in circHIPK3 expression is also worth exploring.

## Figures and Tables

**Table 1:** PCR primer sequence, siRNA sequence, shRNA target sequence, and FISH probe sequence.

Primer sequence	
circHIPK3	F: 5′-GGATCGGCCAGTCATGTATC-3′
	R: 5′-ACCGCTTGGCTCTACTTTGA-3′

miR-29a	RT: GTCGTATCCAGTGCGTGTCGTGGAGTCGGCAATTGCACTGGATACGACTAACCGAT
	F: ACACTCCAGCTGGGTAGCACCATCTGAAAT
	R: TGGTGTCGTGGAGTCG

U6	RT: 5′-AACGCTTCACGAATTTGCGT-3′
	F: 5′-CTCGCTTCGGCAGCACA-3′
	R: 5′-AACGCTTCACGAATTTGCGT-3′

IGF-1	F: 5′-CATCTCTTCTACCTGGCACTCTG-3′
	R: 5′-TTGGTCCACACACGAACTGAA-3′

GAPDH	F: 5′-GTCAAGGCTGAGAACGGGAA-3′
	R: 5′-AAATGAGCCCCAGCCTTCTC-3′

*β*-Actin	F: 5′-TTGTTACAGGAAGTCCCTTGCC-3′
	R: 5′-ATGCTATCACCTCCCCTGTGTG-3′

siRNA sequence	circHIPK3 siRNA 5′-TAGAAGACCATGGGGGATA-3′
	linearHIPK3 siRNA 5′-GCUGAUUGAUGCAGAUUUA-3′

FISH probe:	
circHIPK3	5′-TGGGTAGACCAAGACTTGTGAGGCCATACCTGTAGTACCGAGA-3′
miR-29a	5′-TAACCGATTTCAGATGGTGCTA-3′

## Data Availability

The data used to support the findings of this study are included within the article.
